# Small RNA profiling of low biomass samples: identification and removal of contaminants

**DOI:** 10.1186/s12915-018-0522-7

**Published:** 2018-05-14

**Authors:** Anna Heintz-Buschart, Dilmurat Yusuf, Anne Kaysen, Alton Etheridge, Joëlle V. Fritz, Patrick May, Carine de Beaufort, Bimal B. Upadhyaya, Anubrata Ghosal, David J. Galas, Paul Wilmes

**Affiliations:** 1 Luxembourg Centre for Systems Biomedicine, University of Luxembourg, 4362 Esch-sur-Alzette, Luxembourg; 2 Present address: German Centre for Integrative Biodiversity Research (iDiv) Leipzig-Halle-Jena, 04103 Leipzig, Germany; 3 Department of Soil Ecology, Helmholtz-Centre for Environmental Research GmbH (UFZ), 06120 Halle (Saale), Germany; 4 Present address: Dilmurat Yusuf, Bioinformatics Group, Department of Computer Science, University of Freiburg, 79110 Freiburg, Germany; 5 Present address: Centre Hospitalier de Luxembourg, 1210 Luxembourg, Luxembourg; 6 Pacific Northwest Research Institute, Seattle, WA 98122 USA; 7 Present address: Department of Biology, Massachusetts Institute of Technology, Cambridge, MA 02139 USA

**Keywords:** RNA sequencing, Artefact removal, Exogenous RNA in human blood plasma, Contaminant RNA, Spin columns

## Abstract

**Background:**

Sequencing-based analyses of low-biomass samples are known to be prone to misinterpretation due to the potential presence of contaminating molecules derived from laboratory reagents and environments. DNA contamination has been previously reported, yet contamination with RNA is usually considered to be very unlikely due to its inherent instability. Small RNAs (sRNAs) identified in tissues and bodily fluids, such as blood plasma, have implications for physiology and pathology, and therefore the potential to act as disease biomarkers. Thus, the possibility for RNA contaminants demands careful evaluation.

**Results:**

Herein, we report on the presence of small RNA (sRNA) contaminants in widely used microRNA extraction kits and propose an approach for their depletion. We sequenced sRNAs extracted from human plasma samples and detected important levels of non-human (exogenous) sequences whose source could be traced to the microRNA extraction columns through a careful qPCR-based analysis of several laboratory reagents. Furthermore, we also detected the presence of artefactual sequences related to these contaminants in a range of published datasets, thereby arguing in particular for a re-evaluation of reports suggesting the presence of exogenous RNAs of microbial and dietary origin in blood plasma. To avoid artefacts in future experiments, we also devise several protocols for the removal of contaminant RNAs, define minimal amounts of starting material for artefact-free analyses, and confirm the reduction of contaminant levels for identification of bona fide sequences using ‘ultra-clean’ extraction kits.

**Conclusion:**

This is the first report on the presence of RNA molecules as contaminants in RNA extraction kits. The described protocols should be applied in the future to avoid confounding sRNA studies.

**Electronic supplementary material:**

The online version of this article (10.1186/s12915-018-0522-7) contains supplementary material, which is available to authorized users.

## Background

The characterisation of different classes of small RNAs (sRNAs) in tissues and bodily fluids holds great promise for understanding human physiology as well as in health-related applications. In blood plasma, microRNAs and other sRNAs are relatively stable, and microRNAs in particular are thought to reflect a system-wide state, making them potential biomarkers for a multitude of human diseases [1, 2]. Different mechanisms of sRNA delivery as a means of long-distance intercellular communication have been recognised in several eukaryotes [3–10]. In addition, inter-individual, inter-species and even inter-kingdom communications via sRNAs have been proposed [11–15], and cases of microRNA-based control by the host [16, 17] or pathogens [18, 19] have been demonstrated.

Additionally, exogenous RNAs have been reported in the blood plasma of humans and mice [20, 21], sparking a heated debate around the genuineness of these observations [22–25]. While bacteria do secrete RNAs via outer membrane vesicles [26–28], the potential for exogenous RNA-based signalling in mammals is also the subject of significant current debate [29, 30]. Diet-derived exogenous microRNAs have been proposed to exert an influence on human physiology [31, 32], but these findings have been refuted by others due to a lack of reproducibility in validation studies [33–37]. This discussion happens at a time when DNA sequencing-based analyses of low-biomass samples have been recognised as prone to being confounded by contaminants [38]. From initial sample handling [39], to extraction kits [40], to sequencing reagents [41], multiple sources of DNA contamination and artefactual sequencing data have been described.

Herein, we report on the contamination of widely used silica-based columns for the isolation of micro- and other sRNAs with RNA, which was apparent from sRNA sequencing data and was subsequently validated by qPCR. These artefactual sRNA sequences are also apparent in numerous published datasets. Furthermore, approaches for the depletion of the contaminants from the columns as well as an evaluation of a newer ultra-clean kit are presented, along with the determination of a minimum safe input volume to suppress the signal of the contaminant sequences in RNA sequencing data of human blood plasma samples. The potential presence of bona fide exogenous sRNA species in human plasma is examined. Finally, recommendations for the control and interpretation of sRNA sequencing data from low-biomass samples are provided.

## Results

### Initial detection of exogenous sRNAs in human blood plasma

**Fig. 1:**
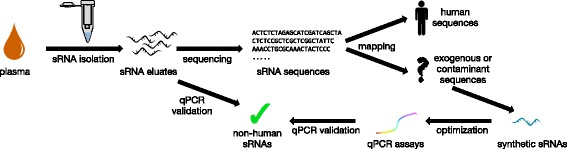
Workflow of the initial screen for and validation of exogenous sRNA sequences in human plasma samples

sRNA was extracted from 100 μL of blood plasma samples of 10 healthy individuals and sequenced using regular RNeasy columns (workflow in Fig. 1). The read profiles were mined for putative exogenous (non-human) sequences (see Methods). Among the potential exogenous sequences were 19 sequences that occurred with more than 1000 counts per million (cpm) in all samples. To rule out sequencing errors or contamination during sequencing library preparation, a qPCR assay was developed to assess the presence of non-human sequences in the sRNA preparations from plasma. Six of the 19 highly abundant sRNA sequences from plasma that could not be mapped to the human genome were chosen for validation by qPCR (Table 1).

**Table 1:** Sequences of non-human sRNAs found in plasma preparations, synthetic sRNA templates, primers and annealing temperatures

Name	RNA sequence	Average counts per million in 10 plasma samples	Potential origin of sequence	Primer sequence	Annealing temperature
sRNA 1	(CU)AACAGACCGAGGACUUGAA(U)	133,700	algae	AACAGACCGAGGACTTGAA	57 °C
sRNA 2	ACGGACAAGAAUAGGCUUCGGCU	8000	fungi or plants	ACGGACAAGAATAGGCTTC	54 °C
sRNA 3	GCCUUGGUUGUAGGAUCUGU	8200	plants	GCCTTGGTTGTAGGATCTGT	57 °C
sRNA 4	GCCAGCAUCAGUUCGGUGUG	6800	bacteria	CAGCATCAGTTCGGTGTG	57 °C
sRNA 5	GAGAGUAGGACGUUGCCAGGUU	3900	bacteria	AGTAGGACGTTGCCAGGTT	57 °C
sRNA 6	UUGAAGGGUCGUUCGAGACCAGGACGUUGAUAGGCUGGGUG	3400	bacteria	GAAGGGTCGTTCGAGACC	57 °C
*hsa*-miR486-5p	UCCUGUACUGAGCUGCCCCGAG		human	–*	60 °C

* *hsa-miR486-5p* specific assay from Quanta BIOSCIENCES

### qPCR assays for putative exogenous sRNAs in human blood plasma

**Fig. 2:**
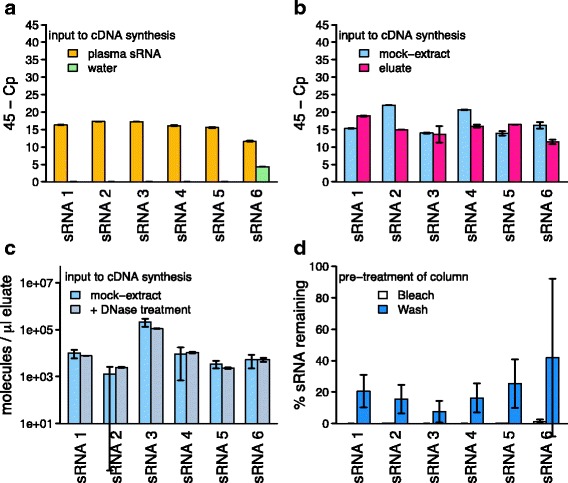
Detection of non-human sRNA species in column eluates and their removal from columns: **a** qPCR amplification of six non-human sRNA species in extracts from human plasma and qPCR control (water). **b** Detection of the same sRNA species in mock extracts without input to extract columns and water passed through extraction columns (‘eluate’). **c** Levels of the same sRNA species in mock extracts without and with DNase treatment during the extraction. **d** Relative levels of sRNA remaining after pre-treatment of extraction columns with bleach or washing ten times with water, detected after eluting columns with water. All: mean results of three experiments, measured in reaction duplicates; error bars represent one standard deviation; data points are available in Additional file 2: Tables S7–S10. Experiments displayed in panels **b** and **d** were performed on the same batch of columns, **a** and **c** on independent batches

Synthetic sRNAs with the putative exogenous sequences found in plasma were poly-adenylated and reverse transcribed to yield cDNA, and used for optimisation of PCR primers and conditions (Table 1). All primer sets yielded amplicons with single peaks in melting temperature analysis with efficiency values above 80%. The optimised qPCR assays were then employed to test for the presence of the highly abundant sRNAs potentially representing exogenous sequences (workflow in Fig. 1) in the human plasma samples used for the initial sequencing experiment. The qPCR assays confirmed the presence of these sRNAs in the sRNA preparations used for sequencing (Fig. 2a), yielding amplicons with melting temperatures expected from the synthetic sRNAs. No amplification was observed if the poly-adenylation or the reverse transcription step were omitted. To rule out contamination of the water used in the sRNA preparations, a water control was also examined. No amplification was observed in all but one assay, where amplification of a product with a different melting temperature occurred (Fig. 2a). Thus, for the assays, water contamination could be ruled out.

### Non-human sequences derived from column contaminants

To analyse whether the validated non-human sequences occurring in the sRNA extracts of plasma were present in any lab-ware, a series of control experiments were carried out (Additional file 1: Figure S1). When nucleic acid- and RNase-free water (QIAGEN) was used as input to the miRNeasy Serum/Plasma kit (QIAGEN) instead of plasma (‘mock extraction’), all tested non-human sequences could be amplified from the mock extract (Fig. 2b), indicating that one of the components of the extraction kit or lab-ware was contaminated with the non-human sequences. To locate the source of contamination, mock extractions were performed by omitting single steps of the RNA isolation protocol except for the elution step. Amplification from the resulting mock extracts was tested for the most abundant non-human sequence (sRNA 1). In all cases, the sRNA 1 could be amplified (data not shown). We therefore performed a simple experiment in which nucleic acid- and RNase-free water was passed through an otherwise untreated spin column. From this column eluate, all target sequences could be amplified (Fig. 2b), in contrast to the nucleic acid- and RNase-free water (Fig. 2a). The most abundant non-human sequences in the plasma sequencing experiments were therefore most likely contaminants originating from the RNeasy columns.

### Detection of contaminant sequences in public datasets

**Fig. 3:**
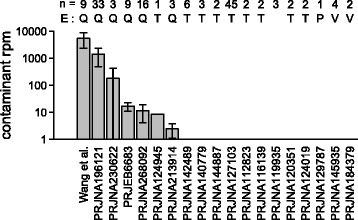
Detection of contaminant sequences in published sRNA sequencing datasets of low biomass samples. Datasets are referenced by NCBI bioproject accession or first author of the published manuscript. *n* number of samples in the dataset, *E* extraction kit used (if this information is available), *Q* regular miRNeasy (QIAGEN), *T* TRIzol (Thermo Fisher), *P* mirVana PARIS RNA extraction kit (Thermo Fisher), *V* mirVana RNA extraction kit with phenol, *Rpm* reads per million. Error bars indicate one standard deviation

To assess whether our observation of contaminant sRNAs was also pertinent in other sequencing datasets of low-input samples, the levels of confirmed contaminant sRNA sequences in published datasets [20, 21, 34, 42–59] were assessed. Irrespective of the RNA isolation procedure applied, non-target sequences were detected (making up between 5% and over 99% of the sequencing libraries for the human samples; Additional file 2: Table S2). As shown in Fig. 3, the six contaminant sequences which had been confirmed by qPCR were found in all analysed low biomass samples extracted with regular miRNeasy kits, but the sequences were found at lower levels in studies with more biomass input [34, 43, 45] and hardly ever [46] in studies where samples were extracted using other methods (Additional file 2: Table S2). Within each study where the confirmed contaminant sequences were detected, the relative levels of the contaminant sequences were remarkably stable (Additional file 3: Figure S2).

### Depletion of contaminants from isolation columns

In order to eliminate contamination from the columns to allow their use in studies of environmental samples or potential exogenous sRNAs from human samples, we were interested in the nature of these contaminants. The fact that they can be poly-adenylated by RNA-poly-A-polymerase and need to be reverse-transcribed before amplification indicates to them being RNA. Treatment of the eluate with RNase prior to cDNA preparation also abolished amplification (data not shown), but on-column DNase digestion did not reduce their levels (Fig. 2c). Thus, these findings suggest that the contaminants were RNAs.

Contaminating sequences could potentially be removed from the RNeasy columns using RNase, but as RNases are notoriously difficult to inactivate and RNases remaining on the column would be detrimental to sRNA recovery, an alternative means of removing RNA was deemed desirable. Loading and incubation of RNeasy columns with the oxidant sodium hypochlorite and subsequent washing with RNase-free water to remove traces of the oxidant reduced the amplifyability of unwanted sRNA by at least 100 times (Fig. 2d) while retaining the columns’ efficiency to isolate sRNAs from samples applied afterwards. Elimination of contaminant sRNAs from the RNeasy columns by washing with RNase-free water (Fig. 2d; average ± standard deviation of the contaminant reduction by 80 ± 10%) or treatment with sodium hydroxide (70 ± 15%) was not sufficient to completely remove the contaminants.

### Ultra-clean extraction kits

**Fig. 4:**
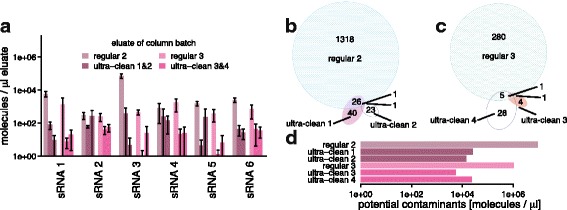
Confirmed and potential contaminant sequences in eluates of regular and ultra-clean RNeasy spin columns: **a** Levels of contaminant sequences in eluates of two batches of regular and four batches of ultra-clean spin columns, based on qPCR; ultra-clean batches 1 and 2 are cleaned-up versions of regular batch 2 and ultra-clean batches 3 and 4 are cleaned-up versions of regular batch 3; error bars indicate one standard deviation; data points are available in Additional file 2: Table S11. **b** and **c** Numbers of different further potential contaminant sequences on the regular and ultra-clean spin columns from two different batches. **d** Total levels of further potential contaminant sequences, based on sRNA sequencing data normalised to spike-in levels. *Cpm* counts per million

Recently, RNeasy columns from an ultra-clean production have become available from QIAGEN within the miRNeasy Serum/Plasma Advanced Kit. We compared the levels of the previously analysed contaminant sequences in the flow-through of mock extractions using four batches of ultra-clean RNeasy columns to two batches of the regular columns by qPCR. In all cases, marked reductions in the contaminant levels were observed in the clean columns (Fig. 4a; 4 to 4000 fold; median 60). To obtain an overview of other potential contaminants, sRNA sequencing of the mock extracts from these six batches of spin columns was performed. With regards to the six previously analysed contaminant sequences, the results were similar to those of the qPCR assays (Additional file 4: Figure S3). Additionally, for the ultra-clean RNeasy columns, a smaller spectrum of other potential contaminant sequences was observed (Fig. 4b, c) and those sequences made up a smaller proportion of the eluate sequences (Fig. 4d).

**Fig. 5:**
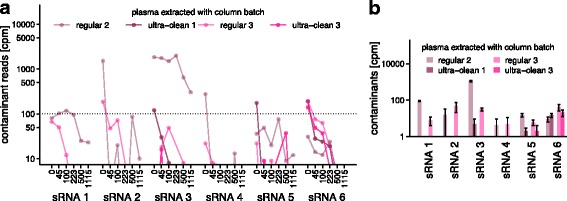
Titration experiment: Detection of contaminants in sRNA preparations of human plasma using different input volumes and extraction columns. **a** Detected levels of the six contaminant sRNA sequences in sRNA sequencing data of preparations using 0 to 1115 μL human plasma and regular or ultra-clean RNeasy spin columns. **b** Detailed view of the data displayed in **a** for 100 μL of human plasma as input to regular and ultra-clean RNeasy spin columns. *Cpm* counts per million. Error bars indicate one standard deviation; data points are available in Additional file 2: Table S12

As our initial analyses of plasma samples extracted using regular RNeasy spin columns had revealed contaminant levels of up to 7000 cpm, we were interested to define a safe input amount for human plasma for both column types that would be sufficient to suppress the contaminant signals to below 100 cpm. For this, we performed a titration experiment (Additional file 4: Figure S3b), isolating sRNA from a series of different input volumes of the same human plasma sample on four batches of RNeasy columns (two batches of regular columns, two batches of ultra-clean columns) with subsequent sequencing. As expected from reagent contaminants, the observed levels of the contaminant sequences were generally inversely dependent on the plasma input volume (Fig. 5a). In addition, and in accordance with the earlier mock extraction results, the levels of contaminant sequences were lower or they were completely absent in the ultra-clean columns (see levels for 100 μL input in Fig. 5b). An input volume of 100 μL of plasma was sufficient to reduce all contaminant sequences to below 100 cpm when using the ultra-clean spin columns.

### Potential plasma-derived exogenous RNAs

Finally, to assess whether any potential exogenous sRNAs might be present in human plasma, we mined the plasma datasets used in the well-controlled titration experiment for sequences that did not originate from the human genome or from known contaminants of sequencing reagents and were not detected in any of the mock-extracts. On average, 5% of the sequencing reads of sRNA isolated from plasma did not map to the human genome; 127 sequences which did not map to the human genome assembly hg38 were detected in the majority of the plasma samples and were not represented in the control samples (empty libraries, mock extractions, column eluates or water). Out of these, 3 sequences had low complexity; 81 sequences could be exactly matched to sequences in the NCBI-nr that are not part of the current version of the human genome assembly (hg38) but annotated as human sequences, or had best partial matches to the human genome or to sequences from other vertebrates; and, of the 43 remaining sequences, which matched best to bacterial, fungal or plant sequences, 22 matched best to the genomes of genera that have previously been identified as contaminations of sequencing kits [41] and were removed. The remaining 21 sequences displayed very low relative abundances close to the detection limit (always below 50 cpm, mean below 5 cpm) in the 28 datasets derived from a single plasma sample from the one healthy individual (Additional file 5: Figure S4). Their potential origins were heterogeneous, including a plant, fungi and bacteria, with an enrichment in partial or perfect hits to *Lactobacillus* sequences (Additional file 2: Table S2). No signature of dietary or common gut microbial organisms was observed.

## Discussion

Several instances of contamination of laboratory reagents with DNA, which can confound the analysis of sequencing data, have been reported in recent years [38, 41, 60, 61]. In contrast, the contamination of reagents with RNA has not yet been reported. Contamination with RNA is usually considered very unlikely due to the ubiquitous presence of RNases in the environment and RNA’s lower chemical stability given its tendency towards hydrolysis, especially at higher pH. However, our results suggest that the detected contaminants were not DNA, but RNA, because treatment with RNase and not DNase decreased the contaminant load. In addition, the contaminating molecules could not be amplified without poly-adenylation and reverse transcription. The stability of the contaminants is likely due to the extraction columns being RNase-free and their silica protecting bound sRNAs from degradation.

The results presented here focused on one manufacturer’s spin column-based extraction kit, which is commonly used in studies on samples with low RNA content, in particular human blood plasma, on which this kit was used because it was amongst those showing the highest yields in studies comparing different kits [62–65]. However, other RNA-stabilising or extraction reagents may carry RNA contamination. Based on the analysis of the published datasets, where significant numbers of sequences that did not map to the source organism’s genome were found to be independent of the RNA extraction kit used, potential contaminants in other extraction kits would have different sequences than those confirmed by qPCR herein. As suggested by previously observed significant batch effects of sequencing data derived from samples extracted with a number of different extraction kits [24], the contaminants may also qualitatively and quantitatively change over time. It is therefore highly recommended to properly control the different sample handling procedures and RNA isolation steps for contaminants when assessing unexpected RNAs in low biomass samples, independent of the extraction kit.

The methods presented here should also help to re-assess the question of whether exogenous sRNA species derived from oral intake [21] or the microbiome [20, 44, 66] really occur in human plasma or are merely artefacts [23]. The limited data source from this study (one healthy person) points to very low levels and a small spectrum of potential foreign sRNAs without an obvious link to diet and which may have been introduced during venipuncture, which is impossible to control for. Additional data from a large number of subjects will be required to make any conclusive statements in this context.

The reported contaminant sequences can confound studies of organisms whose transcriptomes contain sequences similar to the contaminants. While they are not abundant enough to confound biomarker studies in human plasma by dilution effects, they may lead to the overestimation of miRNA yields in low-biomass samples. They can also give rise to misinterpretation in studies without a priori knowledge of the organisms present.

## Conclusions

**Fig. 6:**
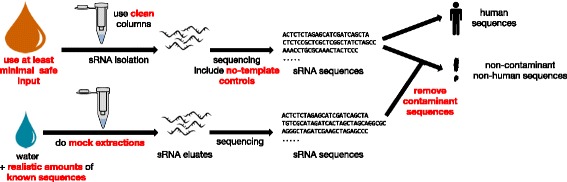
Summary: Recommendations for artefact-free analysis of sRNA by sequencing

Care has to be taken when analysing low-input samples, in particular for surveys of environmental or otherwise undefined sources of RNAs. A number of recommendations can be conceived based on the presented data (Fig. 6). First, extraction columns should be obtained as clean as possible. Second, simple clean-up procedures can also reduce contaminants. Third, the input mass of sRNA should be as high as possible, e.g. for human plasma, volumes above 100 μL are preferable. Fourth, extraction controls should always be sequenced with the study samples. To facilitate library preparation for the extraction controls, spike-in RNAs with defined sequences can be used and should be applied at concentrations similar to the levels of RNA found in the study samples. As the spike-in signal can drown out the contaminants, it is necessary to avoid concentrations that are too high for the spike-ins. Fifth, sequences found in the extraction controls should be treated as artefacts and removed from the sequencing data. Independent techniques that are more robust to low input material, such as qPCR or ddPCR, should be applied to both study samples and controls in case of doubt.

## Methods

### Blood plasma sampling

Written informed consent was obtained from all blood donors. The sample collection and analysis was approved by the Comité d’Ethique de Recherche (CNER; Reference: 201110/05) and the National Commission for Data Protection in Luxembourg. Blood was collected by venepuncture into EDTA-treated tubes. Plasma was prepared immediately after blood collection by centrifugation (10 min at 1000 × *g*) and platelets were depleted by a second centrifugation step (5 min at 10,000 × *g*). The blood plasma was flash-frozen in liquid nitrogen and stored at −80 °C until extraction.

### Use of sRNA isolation columns

Unless stated otherwise, 100 μL of blood plasma was lysed using the QIAzol (QIAGEN) lysis reagent prior to binding to the column, as recommended by the manufacturer. RNeasy MinElute spin columns from the miRNeasy Serum/Plasma Kit (QIAGEN) were then loaded, washed and dried, and RNA was eluted as recommended by the manufacturer’s manual. We further tested four batches of ultra-clean RNeasy MinElute columns, which underwent an ultra-clean production process to remove potential nucleic acid contamination, including environmental sRNAs. These columns were treated as recommended in the manual of the miRNeasy Serum/Plasma Advanced Kit (QIAGEN). All eluates were stored at −80 °C until analysis.

For the mock extractions, ultra-clean or regular RNeasy columns were loaded with the aqueous phase from a QIAzol extraction of nucleic acid- and RNase-free water (QIAGEN) instead of plasma. For mock extractions with a defined spike-in, the aqueous phase was spiked with synthetic *hsa*-miR-486-3p RNA (Eurogentec) to yield 40,000 copies per μL of eluate. To obtain column eluates, spin columns were not loaded, washed or dried. Instead, 14 μL of RNase-free water (QIAGEN) was applied directly to a new column and centrifuged for 1 min. In the plasma titration experiment, plasma input volumes of 45, 100, 225, 500, and 1115 μL and 100 μL of RNase-free water that had been pre-processed analogously to the plasma samples were used for the QIAzol (QIAGEN) step.

To eliminate environmental sRNAs from the regular RNeasy columns, the columns were incubated with 500 μL of a sodium hypochlorite solution (Sigma; diluted in nuclease free water (Invitrogen) to approx. 0.5%) for 10 min at room temperature. Columns were subsequently washed 10 times with 500 μL of nuclease free water (Invitrogen), before use. Similarly, in the attempt to remove sRNAs by application of sodium hydroxide, 500 μL of 50 mM NaOH were incubated on the spin columns for 5 min, followed by incubation with 50 mM HCl for 5 min, prior to washing the columns 10 times with 500 μL of nuclease-free water (Invitrogen) before use.

### Real-time PCR

Eluted RNA (5 μL) was polyadenylated and reverse-transcribed to cDNA using the qScript microRNA cDNA Synthesis Kit (Quanta BIOSCIENCES). cDNA (1 μL, except for the initial plasma experiment, where 0.2 μL of cDNA were used) was amplified by use of sequence-specific forward primers (see Table 1, obtained from Eurogentec) or the miR486-5p-specific assay from PerfeCTa Universal PCR Primer and PerfeCTa SYBR Green SuperMix (Quanta BIOSCIENCES) in a total reaction volume of 10 μL. Primers were added at a final concentration of 0.2 μM. Primer design and amplification settings were optimised with respect to reaction efficiency and specificity. Efficiency was calculated using a dilution series covering seven orders of magnitude of template cDNA reverse transcribed from synthetic sRNA. Real-time PCR was performed on a LightCycler^®^ 480 Real-Time PCR System (Roche) including denaturation at 95 °C for 2 min and 40 cycles of 95 °C for 5 s, 54–60 °C for 15 s (for annealing temperatures see Table 1), and 72 °C for 15 s. All reactions were performed in duplicate. No-template controls were performed analogously with water as input. Controls without reverse transcriptase were performed with the mock extract experiments and did not yield amplicons. Cp values were obtained using the second derivative procedure provided by the LightCycler^®^ 480 Software, Version 1.5. Absolute quantification of sRNAs in the eluates was enabled by the dilution series of defined concentrations of synthetic sRNAs with the same sequence as the target sRNAs. Linear regression of the C_T_ against the log_10_ concentration was performed to yield the intercept *b* and slope *m*, which were used to calculate the number of sRNAs in the test samples 10^(*b* – CT/–*m*)^.

### sRNA seq: library preparation and sequencing

sRNA libraries were made using the TruSeq small RNA library preparation kit (Illumina) according to the manufacturer’s instructions, except that the 3′ and 5′ adapters were diluted 1:3 before use. PCR-amplified libraries were size selected using a PippinHT instrument (Sage Science), collecting the range of 121 to 163 bp. Completed, size-selected libraries were run on a High Sensitivity DNA chip on a 2100 Bioanalyzer (Agilent) to assess library quality. Concentration was determined by qPCR using the NEBNext Library Quant kit. Libraries were pooled, diluted and sequenced with 75 cycle single-end reads on a NextSeq 500 (Illumina) according the manufacturer’s instructions. The sequencing reads can be accessed at NCBI’s short read archive via PRJNA419919 (for sample identifiers and accessions see Additional file 2: Table S1).

### Initial analysis: plasma-derived sRNA sequencing data

For the initial analysis of plasma-derived sRNA sequencing data, FastQC [67] was used to determine over-represented primer and adapter sequences, which were subsequently removed using cutadapt [68]. This step was repeated recursively until no over-represented primer or adapter sequences were detected. 5’-Ns were removed using fastx_clipper of the FASTX-toolkit. Trimmed reads were quality-filtered using fastq_quality_filter of the FASTX-toolkit (with -q 30 -p 90) [69]. Finally, identical reads were collapsed, retaining the read abundance information using fastx_collapser of the FASTX-toolkit. The collapsed reads were mapped against the human genome (GRCh37), including RefSeq exon junction sequences, as well as prokaryotic, viral, fungal, plant and animal genomes from GenBank [70] and the Human Microbiome Project [71] using Novoalign V2.08.02 (Additional file 2: Tables S3 to S5) [72]. These organisms were selected based on their presence in the human microbiome, human nutrition and the public availability of the genomes. As reads were commonly mapping to genomic sequences of multiple organisms, and random alignment can easily occur between short sequences and reference genomes, the following approach was taken to refine their taxonomic classification. First, reads were attributed to the human genome if they mapped to it. Secondly, reads mapping to each reference genome were compared to mapping of a shuffled decoy read set. Based on this, the list of reference genomes was limited to the genomes recruiting at least one read with a minimum length of 25 nt. Loci on non-human genomes were established by the position of the mapping reads. The number of mapping reads per locus was adjusted using a previously established cross-mapping correction [73]. Finally, the sequences of the loci, the number of mapping reads and their potential taxonomy were extracted.

### sRNA sequence analysis of controls

For the subsequent analysis of the mock extractions, column eluates, and nucleic acid- and RNase-free water, as well as of no-template controls and human plasma samples, extracted using either regular or ultra-clean RNeasy columns, the trimming and quality check of the reads was performed analogously to the description above. Collapsed reads were mapped against the most recent version of the human genome (hg38) either to remove operator-derived sequences or to distinguish the reads mapping to the human genome in the different datasets. Sequencing was performed in two batches, with one batch filling an entire flow cell, and one mixed with other samples. The latter batch of samples was sequenced on the same flow cell as sRNAs extracted from *Salmonella typhimurium* LT2. To avoid misinterpretations due to multiplexing errors, reads mapping to *Salmonella typhimurium* LT2 [74] (GenBank accession AE006468) were additionally removed in this batch. To limit the analysis to only frequently occurring sequences and therefore avoid over-interpretation of erroneous sequences, only read sequences that were found at least 30 times in all analysed samples together were retained for further analysis. Public sRNA datasets of low-input samples (Additional file 2: Table S1) were analysed in a fashion analogous to the study’s control and plasma samples. As the published studies consisted of different numbers of samples, no overall threshold was imposed, but to limit the analysis to frequently occurring sequences, singleton reads were removed.

To compare the sequencing results to the qPCR-based results and to detect the same sequences in public datasets, reads matching the sequences assayed by qPCR were determined by clustering the trimmed, filtered and collapsed sRNA reads with 100% sequence identity and 14 nt alignment length with the primer sequences, while allowing the sRNA reads to be longer than the primer sequences, using CD-HIT-EST-2D (parameters -c 1 -n 8 -G 0 -A 14 -S2 40 -g 1 -r 0) [75].

To compare the diversity and levels of putative contaminant sequences in the different samples, identical reads derived from all study samples (that did not map to the human genome) were clustered using CD-HIT-EST [75], and a table with the number of reads sequenced for each sample per sequence was created using R v.3.0.2. To obtain estimates of absolute numbers of contaminant sequences, the cpm of non-human sequences were normalised to the cpm of the spike-in *hsa-*miR-486-5p, whose abundance was determined both from the sequencing as well as the qPCR experiments.

The table of counts of identical sequences per sample was also used to extract candidate sequences from the study plasma samples that are likely exogenous plasma sRNAs, based on the following criteria: for a sequence to be considered a potential exogenous plasma sRNA, it had to be non-identical to any of the sequences assigned to the confirmed contaminant sequences (Table 1), it had to be absent from at least 90% of the controls (no-library controls, water and spike-in controls, eluates and mock extracts) and never detected in any of these controls with at least 10 copy numbers, and it had to be detected by more than 3 reads in more than 7 of the 28 libraries generated from the plasma titration experiment. These thresholds were chosen in order to make the analysis robust against multiplexing errors (e.g. which would result in false-negative identifications if a sequence that is very dominant in a plasma sample is falsely assigned to the control samples), while at the same time making it sensitive to low-abundant sequences (which would not be detected in every library). To confirm the non-human origin and find potential microbial taxa of origin for these sequences, they were subsequently searched within the NCBI nr database using megablast and blastn web tools, with parameters auto-set for short inputs [76–78]. All sequences with best hits to human sequences or other vertebrates were removed because they were potentially human. The remaining sequences were matched against a set of genera previously reported to be common sequencing kit contaminants [41]. Sequences with better hits to non-contaminant than contaminant taxa were kept as potential exogenous sequences.

## Additional files

Additional file1: **Figure S1.** Scheme summarising the different control experiments, the titration experiments and their outcomes. a) Tracing non-human sRNA sequences to contaminants on spin columns by variation of different steps in the isolation protocol and analysis by qPCR assays. Modifications to the steps named at the top are listed below the workflow and the outcomes are summarised at the right hand side. b) Workflow of the titration experiment to determine a minimal safe input volume for all contaminant sequences. *UCP column* ultra-clean column. (PDF 86 kb)

Additional file 2: **Table S1.** List of the generated datasets with public accession numbers. **Table S2.** Analysed published datasets with references and public accession numbers. **Table S3.** Potential exogenous sRNA sequences detected in human plasma after removal of contaminants. **Table S4.** List of the prokaryotic species whose reference genomes were used in the initial analysis. **Table S5.** List of the eukaryotic species whose reference genomes and/or cDNA collections were used in the initial analysis. **Table S6.** List of the viruses whose reference genomes were used in the initial analysis. **Table S7.** Data points for Fig. 2a. **Table S8.** Data points for Fig. 2b. **Table S9.** Data points for Fig. 2c. **Table S10.** Data points for Fig. 2d. **Table S11.** Data points for Fig. 4a. **Table S12.** Data points for Fig. 5b. (XLSX 228 kb)

Additional file 3: **Figure S2.** Detection of contaminants in published datasets. Heatmap showing the relative abundances of the confirmed contaminant sequences in published sRNA sequencing data of low-biomass samples. Only samples for which any of the confirmed contaminants were detected are shown. Extraction methods: *Q* regular QIAGEN miRNeasy; *T* TRIZOL. *rpm* reads per million. (PDF 106 kb)

Additional file 4: **Figure S3.** Detection of contaminants in eluates of regular and ultra-clean RNeasy columns. Two batches of regular miRNeasy columns and four batches of ultra-clean RNeasy columns were compared. Results are based on sRNA sequencing data of mock extracts, normalised to the detected levels of spike-in synthetic RNAs. The different shadings represent reads mapping to the human genome with 2, 1, or 0 mismatches and the different column batches are coloured in the same colours as in main Fig. 3, as indicated in the legends. (PDF 16 kb)

Additional file 5: **Figure S4.** Relative abundance of potential exogenous sRNAs in datasets derived from a plasma sample of one healthy individual. Detected levels of the 21 potential exogenous sRNA sequences in preparations using 45 to 1115 μL human plasma and regular or ultra-clean RNeasy spin columns and in controls without plasma, including no library, mock extractions and water controls (*n* = 33). *cpm* counts per million. Error bars indicate one standard deviation; data points are available in Additional file 2: Table S11. (PDF 11 kb)

## Abbreviations

qPCR: real-time quantitative polymerase chain reaction
sRNA: small RNA
