# Supplementary figures and images for: Small RNA profiling of low biomass samples: identification and removal of contaminants
Source: BMC Biol. 2018 May 14;16:52. doi: 10.1186/s12915-018-0522-7 (PMC5952572; doi:10.1186/s12915-018-0522-7)

**A**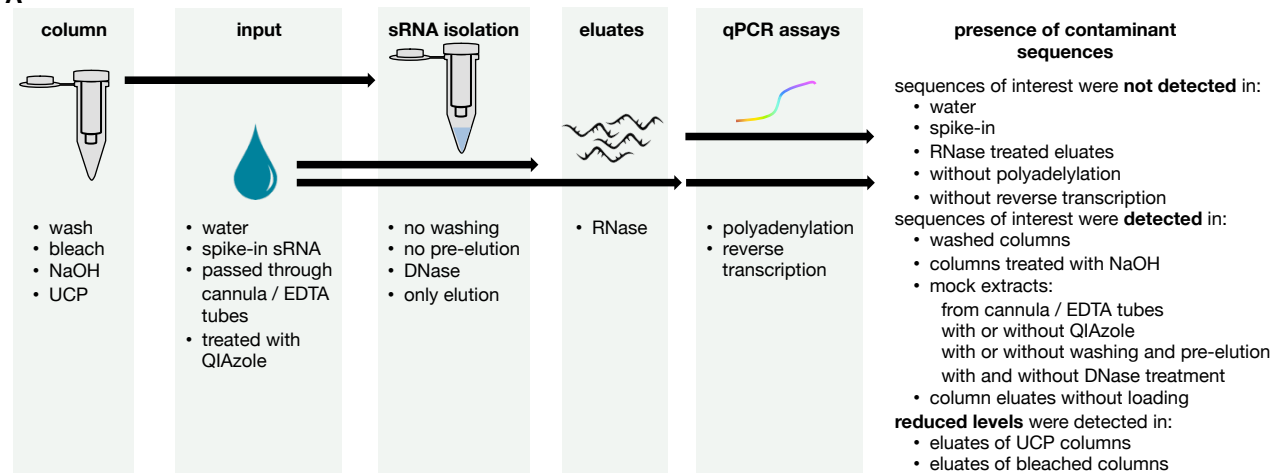**B**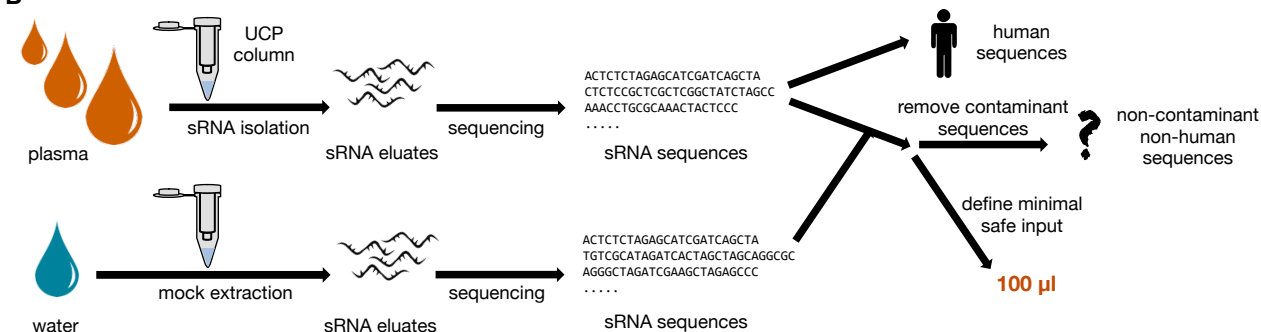

Supplement: Supplementary file 1 — Figure S1. Scheme summarising the different control experiments, the titration experiments and their outcomes. a) Tracing non-human sRNA sequences to contaminants on spin columns by variation of different steps in the isolation protocol and analysis by qPCR assays. Modifications to the steps named at the top are listed below the workflow and the outcomes are summarised at the right hand side. b) Workflow of the titration experiment to determine a minimal safe input volume for all contaminant sequences. UCP column ultra-clean column. (PDF 86 kb) [file 12915_2018_522_MOESM1_ESM.pdf]

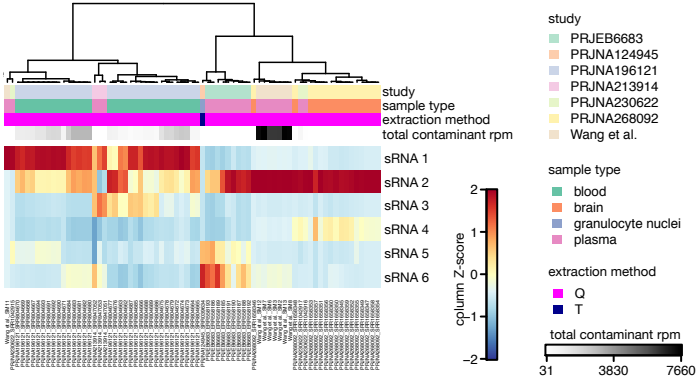

Supplement: Supplementary file 3 — Figure S2. Detection of contaminants in published datasets. Heatmap showing the relative abundances of the confirmed contaminant sequences in published sRNA sequencing data of low-biomass samples. Only samples for which any of the confirmed contaminants were detected are shown. Extraction methods: Q regular QIAGEN miRNeasy; T TRIZOL. rpm reads per million. (PDF 106 kb) [file 12915_2018_522_MOESM3_ESM.pdf]

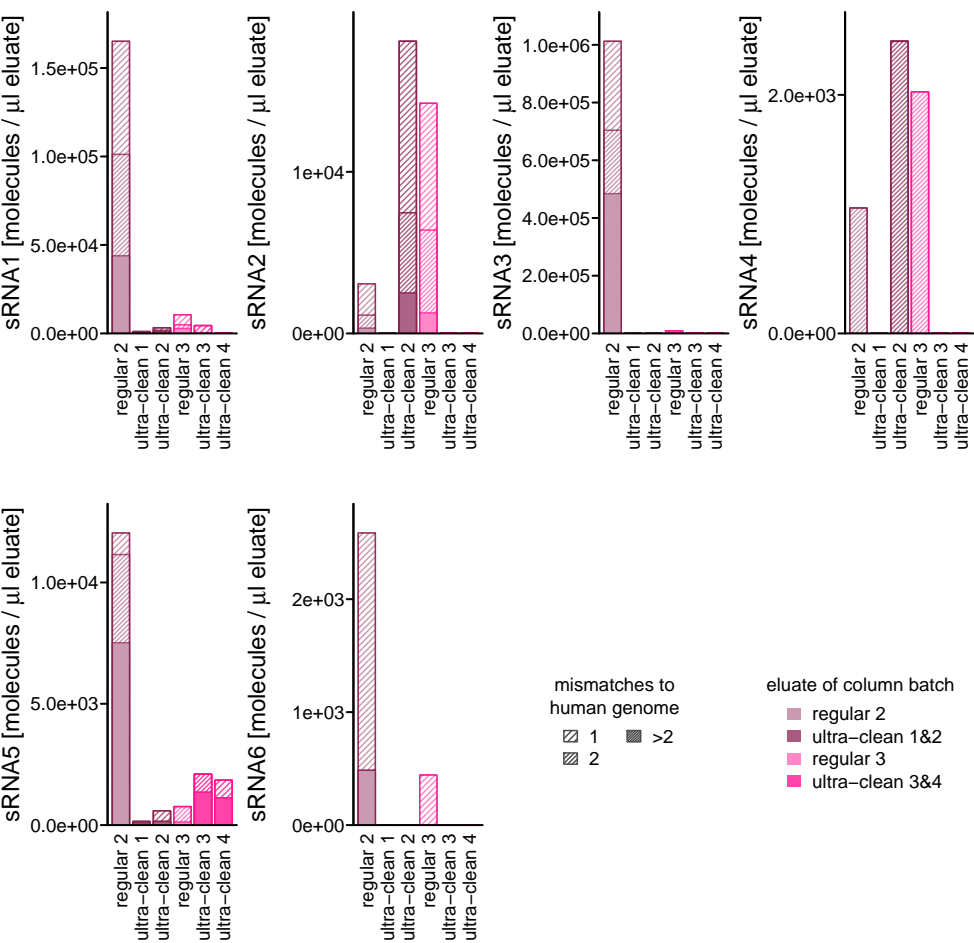

Supplement: Supplementary file 4 — Figure S3. Detection of contaminants in eluates of regular and ultra-clean RNeasy columns. Two batches of regular miRNeasy columns and four batches of ultra-clean RNeasy columns were compared. Results are based on sRNA sequencing data of mock extracts, normalised to the detected levels of spike-in synthetic RNAs. The different shadings represent reads mapping to the human genome with 2, 1, or 0 mismatches and the different column batches are coloured in the same colours as in main Fig. 3, as indicated in the legends. (PDF 16 kb) [file 12915_2018_522_MOESM4_ESM.pdf]

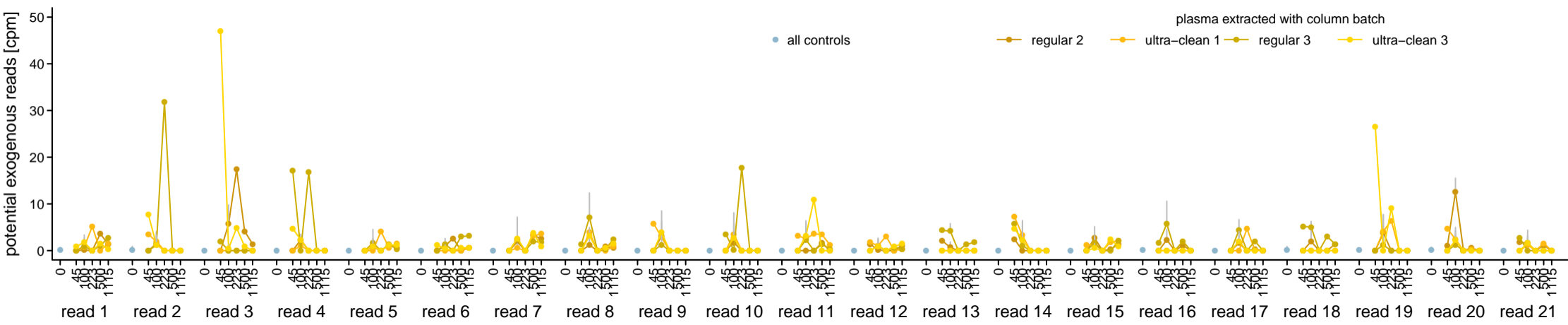

Supplement: Supplementary file 5 — Figure S4. Relative abundance of potential exogenous sRNAs in datasets derived from a plasma sample of one healthy individual. Detected levels of the 21 potential exogenous sRNA sequences in preparations using 45 to 1115 μL human plasma and regular or ultra-clean RNeasy spin columns and in controls without plasma, including no library, mock extractions and water controls (n = 33). cpm counts per million. Error bars indicate one standard deviation; data points are available in Additional file 2: Table S11. (PDF 11 kb) [file 12915_2018_522_MOESM5_ESM.pdf]
